# 

**Published:** 1896-10-31

**Authors:** 


					THE HOSPITAL. ABSOLUTELY PURE, therefore BEST, Ogt. 31st, 189G
CADBURY'8 ^ ti ^ CADBURY'S
COCOA HT* Inl COCOA
'"The standard of highest purity at resent ^ w ? ? - r??
attainable.1 '?L ancet, ^ NO ALKALIES USED.
Thomas s Hosritayyi<11? 11.1 u1>;iiKguuu.'n-i[V
^SODir WES'TERH iNEWiUUf ' " * "* '
No. 5271 Vol. xxi. WITH EXTRA NURSING SUPPLEMENT. pbicb twopence.
o , o_.4.  w ? ? , Per Annum, 10s 6d., post free.
" '?? -?C 111- Oi? If.!,
Saturday,
Oot. 31st, 1896.
[Registered at the General Post Office as a Newspaper for Monthly Parts, 6d. 5 post free, 8d.
transmission in the United Kingdom and Abroad.] Dittoper Annnm,8a. 6d.,pogtfre<j,

				

